# Music therapy effect on anxiety reduction among patients with cancer: A meta-analysis

**DOI:** 10.3389/fpsyg.2022.1028934

**Published:** 2023-01-06

**Authors:** Lu Zang, Chunliang Cheng, Yongxin Zhou, Xuemei Liu

**Affiliations:** ^1^School of Architecture and Art, Central South University, Changsha, Hunan, China; ^2^Department of Urology, National Clinical Research Center for Geriatric Disorders, Xiangya Hospital, Central South University, Changsha, Hunan, China; ^3^School of Music, Theatre and Dance, University of Michigan, Ann Arbor, MI, United States

**Keywords:** music, music therapy, mental health, cancer, meta-analysis

## Abstract

**Introduction:**

The study aimed to investigate the effect of music therapy on anxiety alleviation among cancer patients.

**Methods:**

A comprehensive literature research was performed in four electronic databases (PubMed, Embase, Cochrane Library, and Web of Science). Fifteen randomized controlled trials (RCTs) were included. The risk of bias for the RCTs was evaluated by the Cochrane Risk of Bias tool. Anxiety levels were extracted to synthesize the combined effect by using meta-analysis. All analyses were performed using R version 4.0.4.

**Results:**

In total, 15 RCTs met the inclusion criteria involving 1320 cancer patients (662 patients in the experimental group and 658 patients in the controlled group). The majority of interventions were performed with recorded music lasting for 15-60 minutes. Compared with standard care, music intervention had a moderate superiority of anxiety alleviation (SMD: –0.54, 95% CI: [–0.92, –0.16]).

**Discussion:**

Music intervention could reduce cancer-related anxiety moderately. Nevertheless, the result should be interpreted with caution as high heterogeneity in this pooled study. Well-designed trials with higher quality were still warranted in the future.

## 1. Introduction

Anxiety symptom was remarkably common in cancer survivors. Approximately, 19.0% of patients with cancer suffered from clinical levels of anxiety and 22.6% had subclinical symptoms (Linden et al., 2012). The diagnosis of cancer often tremendously increased the risk of anxiety and impacts patients’ mental health and quality of life (Lee et al., 2021). Clinically, the procedure of cancer diagnosis and treatment also caused anxiety. The discomfort and complication of this procedure would increase the psychological burden. Besides, anxiety in individuals with cancer would increase the possibility of a visit to the emergency department, the length of hospitalizations, and healthcare costs (Mausbach et al., 2020). Thus, patients with cancer with anxiety had a significantly higher risk of cancer-specific mortality and all-cause mortality (Wang et al., 2020). It was important for the clinician to be concerned about anxiety in the routine clinical practice and provide psychosocial support for patients with cancer.

In recent years, several non-pharmacological interventions reported a positive effect in alleviating treatment-related anxiety (Tola et al., 2021). Among them, music therapy was used to relieve depression (Zhao et al., 2016), Alzheimer’s disease (Giovagnoli et al., 2017), and postoperative pain (Simavli et al., 2014). It can be performed in two main forms with the monitoring of a clinician—active and receptive treatments. Active music therapy enabled patients to forwardly participate in the creation of music *via* singing or playing musical instruments. Conversely, receptive music therapy referred to passively listening to live music or recorded music selected by the clinician or the patient. Active music therapy involved multisensory stimulation mainly in the form of teamwork and contributed to patients’ cognition, mood, quality of life, and other aspects (Pacchetti et al., 2000; Wu et al., 2022). By contrast, receptive music therapy was more available and easier to implement in hospitals. The pace and melody of music would distract patients from clinical procedure—most of which were potentially invasive and painful, such as biopsy, operation, chemotherapy, or radiation therapy in the management of cancer.

For these reasons, several randomized controlled trials were carried out to assess the effect of receptive music therapy on anxiety reduction in routine cancer management. To date, the effect of music therapy on anxiety reduction was still inconclusive. The majority of these RCTs demonstrated a positive effect (Shabanloei et al., 2010; Li et al., 2012; Chen et al., 2013), while other RCTs revealed no significant impact on anxiety reduction. The study of Kwekkeboom (2003) showed that the effects of music and standard care, as usual, were equivocal, which was not sufficiently convincing of the limited sample size and diverse clinical procedures. Wren et al. (2019) was a three-arm clinical trial, and the defect of this study was also the limited sample size in each arm. In addition, O’Steen et al. (2021) demonstrated that music therapy did not reduce anxiety to a meaningful degree during radiation therapy. Whereas subsequent RCTs found music therapy a positive effect during radiation therapy (Chen et al., 2013; Rossetti et al., 2017). And meta-analysis review to combine these results is scarce in the literature. Therefore, this study attempted to address this research gap to provide high-quality evidence for this aspect.

## 2. Materials and methods

### 2.1. Search method

The study was conducted in accordance with the Preferred Reporting Items for Systematic Reviews and Meta-Analyses (PRISMA) guidelines (Moher et al., 2009). In May 2022, a comprehensive literature search was performed in four online electronic databases (PubMed, Embase, Cochrane Library, and Web of Science). The start time in the search was restricted to the last 30 years (i.e., 1993–2022). The language of the literature was limited to English. Additionally, available literature from other published meta-analysis were also extracted.

### 2.2. Inclusion criteria and exclusion criteria

The procedure of literature inclusion was in accordance with the PICOS principle, including (1) Population: Adults (≥18 years) who were diagnosed with solid tumors or hematologic malignancies and/or received adjuvant therapy (chemotherapy, radiation therapy, and immunotherapy) in the potential studies; (2) Intervention: The experimental group who received music therapy and routine treatment; (3) Comparison: The controlled group was treated with routine treatment without music therapy; (4) Outcomes: The level of post-test score in anxiety or anxiety score changes which is defined as post-treatment anxiety minus pre-treatment anxiety measured by the State-Trait Anxiety Inventory (STAI); and (5) Study design: Only randomized controlled trial and high-quality quasi-experimental studies were included. We excluded patients who underwent biopsy or operation for diagnostic purposes and those with palliative treatment. Studies exhibiting no post-test score or score change in the full text would also be excluded.

### 2.3. Study selection and data extraction

**FIGURE 1:**
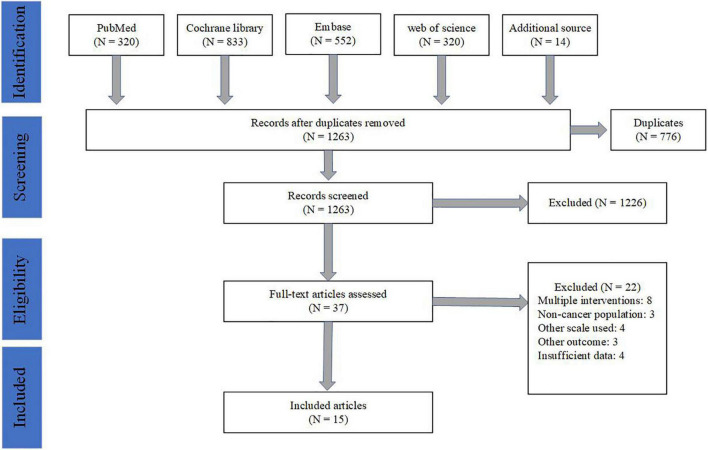
The flow for literature screening.

Two researchers were independently assigned to screen some pieces of literature for identifying potential studies according to the inclusion criteria. Any conflicts in study selection would resort in the third researcher’s judgment. Duplicates were excluded by Endnote X9 software and manual selection. Then researchers screened the title and abstract of passages independently. Any unsuitable types such as reviews, meta-analyses, case reports, short surveys, editorials, letters, laboratory studies, or articles not related to the inclusion criteria would be further excluded. Finally, the full-text articles were obtained and evaluated for final determination. The detailed flow for literature screening is shown in Figure 1. Notably, we included Danhauer et al. with caution for the consideration that bone marrow biopsy in this trial was not performed for diagnostic purposes but served as a routine monitor of treatment.

The general information for the included studies was extracted using a predesigned Excel sheet, which included the first authors’ name, publication year, country, patients’ age, gender distribution, and sample size. Cancer type, intervention in the experimental group, treatment in the controlled group, the procedure and duration of music therapy, scale for outcome measurement, and study result were also extracted.

### 2.4. Risk of bias assessment

The quality of the included literature was assessed by the Cochrane Risk of Bias tool (Higgins et al., 2011). Two researchers independently evaluated the potential bias of RCTs in the seven aspects: “random sequence generation,” “allocation concealment,” “blinding of participants and personnel,” “blinding of outcome assessment,” “incomplete outcome data,” “selective reporting,” and “other bias.” According to the full-text articles, researchers marked these aspects with low, high, or unclear risk of bias. If there were any disagreements in bias assessment, they would be discussed with the third researcher.

### 2.5. Data analysis

The present analysis was performed to identify the effect of music therapy on alleviating cancer-related anxiety. The level of the post-test score and score change were continuous variables given as means ± standard deviations (SD). Cochran’s *Q* test was used to identify the heterogeneity of results by I square (*I*^2^ < 25%: no heterogeneity; *I*^2^ = 25–50%: moderate heterogeneity; *I*^2^ > 50%: large heterogeneity). If *I*^2^ < 50%, the fixed effects model was applied. The random-effects model was adopted if *I*^2^ ≥ 50%, which indicated homogeneous results between included studies (Higgins and Thompson, 2002). Publication bias was visualized by funnel plot. Sensitivity analysis was made by reducing one literature at a time until the heterogeneity was low. A two-sided *P*-value of <0.05 was considered as the threshold of statistical significance. All analysis and figure generation were performed using R version 4.0.4.

## 3. Results

### 3.1. Study characteristics

In total, 15 RCTs (Kwekkeboom, 2003; Bulfone et al., 2009; Danhauer et al., 2010; Lin et al., 2011; Li et al., 2012; O’Callaghan et al., 2012; Chen et al., 2013; Vachiramon et al., 2013; Zhou et al., 2015; Alam et al., 2016; Firmeza et al., 2017; Rossetti et al., 2017; Wren et al., 2019; Al-Jubouri et al., 2021; Mishra et al., 2022) were included in the present analysis involving 1,320 patients with cancer (Table 1). Among these trials, the majority of them (7/15) were conducted in America (Kwekkeboom, 2003; Danhauer et al., 2010; Vachiramon et al., 2013; Alam et al., 2016; Rossetti et al., 2017; Wren et al., 2019; Mishra et al., 2022), followed by four in China (Lin et al., 2011; Li et al., 2012; Chen et al., 2013; Zhou et al., 2015), one in Italy (Bulfone et al., 2009), one in Iraq (Al-Jubouri et al., 2021), one in Australia (O’Callaghan et al., 2012), and one in Brazil (Firmeza et al., 2017). Four studies included patients who had been diagnosed with breast cancer (Bulfone et al., 2009; Li et al., 2012; Zhou et al., 2015; Wren et al., 2019), two studies included patients with skin cancer (Vachiramon et al., 2013; Alam et al., 2016), one study included patients with prostate cancer (Mishra et al., 2022), one study included hematological malignancies (Danhauer et al., 2010), and one study included patients with head and neck cancer (Firmeza et al., 2017). For the rest of the studies, mixed group of cancer patients were included (Kwekkeboom, 2003; Lin et al., 2011; O’Callaghan et al., 2012; Chen et al., 2013; Rossetti et al., 2017; Al-Jubouri et al., 2021). Of all the selected trials, 662 patients underwent music therapy with routine treatment, while 658 patients received only the routine treatment. Three studies (Table 2) were performed to decrease chemotherapy-related anxiety (Bulfone et al., 2009; Lin et al., 2011; Al-Jubouri et al., 2021). Three were performed for radiotherapy-related anxiety alleviation (O’Callaghan et al., 2012; Chen et al., 2013; Rossetti et al., 2017). Eight were designed for mitigating anxiety during surgery (Kwekkeboom, 2003; Danhauer et al., 2010; Li et al., 2012; Vachiramon et al., 2013; Zhou et al., 2015; Alam et al., 2016; Wren et al., 2019; Mishra et al., 2022). Five trials demonstrated its study results with anxiety changes (O’Callaghan et al., 2012; Chen et al., 2013; Alam et al., 2016; Firmeza et al., 2017; Rossetti et al., 2017), while 11 trials only exhibited post-treatment anxiety in the full text (Kwekkeboom, 2003; Bulfone et al., 2009; Danhauer et al., 2010; Lin et al., 2011; Li et al., 2012; O’Callaghan et al., 2012; Vachiramon et al., 2013; Zhou et al., 2015; Wren et al., 2019; Al-Jubouri et al., 2021; Mishra et al., 2022). All the outcomes were measured by STAI.

**TABLE 1:** The characteristics of included studies.

References	Country	Age (mean)	Gender	Size	Cancer type	Experimental	Control	Outcome
Kwekkeboom (2003)	America	I: 51.96; C:53.30	I: 9/15; C: 7/13	I: 24; C: 20	Mixed cancers	Music	Standard care	Post-test score
Bulfone et al. (2009)	Italy	I: 49.2; C: 52.7	F	I: 30; C:30	Breast cancer	Music	Standard care	Post-test score
Danhauer et al. (2010)	America	I: 51.6; C: 50.2	I: 14/16; C: 19/10	I: 29; C: 30	Hematological malignancies	Music	Standard care	Post-test score
Li et al. (2012)	China	I: 44.88; C: 45.13	F	I: 60; C: 60	Breast cancer	Music	Standard care	Post-test score
Lin et al. (2011)	China	I: 50.2; C: 54.3	I: 21/13; C: 23/11	I: 34; C: 34	Lung and breast cancer	Music	Standard care	Post-test score
O’Callaghan et al. (2012)	Australia	I: 58; C: 57	I: 29/21; C: 30/20	I: 50; C: 50	Mixed solid cancer	Music	Standard care	Post-test score and score change
Lin et al. (2011)	China	I: 55.06; C: 55.66	I: 64/36; C: 57/43	I: 100; C: 100	Mixed solid cancer	Music	Standard care	Score change
Vachiramon et al. (2013)	America	I: 62.6; C: 66.0	I: 34/16; C: 33/17	I: 50; C: 50	Skin cancer	Music	Standard care	Post-test score
Zhou et al. (2015)	China	I: 46.80; C: 47.13	F	I: 85; C: 85	Breast cancer	Music	Standard care	Post-test score
Alam et al. (2016)	America	I: 62.4; C: 54.2	I: 32/22; C: 31/20	I: 54; C: 51	Skin cancer	Music	Standard care	Score change
Firmeza et al. (2017)	Brazil	Unknown	20% M; 80.0% F	I: 20; C: 20	Head and neck cancer	Music	Standard care	Score change
Rossetti et al. (2017)	America	I: 58; C: 60	I: 15/24; C: 12/27	I: 39; C: 39	Breast and head and neck cancer	Music	Standard care	Score change
Wren et al. (2019)	America	I: 57.31; C: 52.35	F	I: 16; C: 17	Breast cancer	Music	Standard care	Post-test score
Al-Jubouri et al. (2021)	Iraq	I: 43.42; C: 47.17	I: 31/22; C: 27/26	I: 53; C: 53	Mixed cancers	Music	Standard care	Post-test score
Mishra et al. (2022)	America	I: 64.88; C: 62.13	M	I: 20; C: 20	Prostate cancer	Music	Standard care	Post-test score

I, intervention group; C, controlled group; M, men; W, women.

**TABLE 2:** The details of music intervention.

References	Music type	Play mode	Duration (min)	Frequency	Clinical procedure
Kwekkeboom (2003)	Researcher-selected	Recorded	NA	Once a day	Biopsy, port placement, removal
Bulfone et al. (2009)	Researcher-selected	Recorded	15	Once a day	Chemotherapy
Danhauer et al. (2010)	Researcher-selected	Recorded	NA	Once a day	Bone marrow biopsy
Li et al. (2012)	Patient-preferred	Recorded	30	Twice a day	Mastectomy
Lin et al. (2011)	Patient-preferred	Recorded	60	Once a day	Chemotherapy
O’Callaghan et al. (2012)	Patient-preferred	Recorded	NA	Once a day	Radiotherapy
Lin et al. (2011)	Patient-preferred	Recorded	15	Once a day	Radiotherapy
Vachiramon et al. (2013)	Patient-preferred	Recorded	15–60	Once a day	Mohs surgery
Zhou et al. (2015)	Patient-preferred	Recorded	30	Twice a day	Radical mastectomy
Alam et al. (2016)	Researcher-selected	Recorded	NA	Once a day	Excisional surgery
Firmeza et al. (2017)	Researcher-selected	Recorded	30	Once a day	Outpatient care
Rossetti et al. (2017)	Patient-preferred	Live	20	Once a day	Radiotherapy
Wren et al. (2019)	Patient-preferred	Recorded	20	At least once a day	Breast cancer surgery
Al-Jubouri et al. (2021)	Researcher-selected	Recorded	20	Once a day	Chemotherapy
Mishra et al. (2022)	Patient-preferred	Recorded	30	Once a day	Prostatectomy

### 3.2. Risk of bias

**FIGURE 2:**
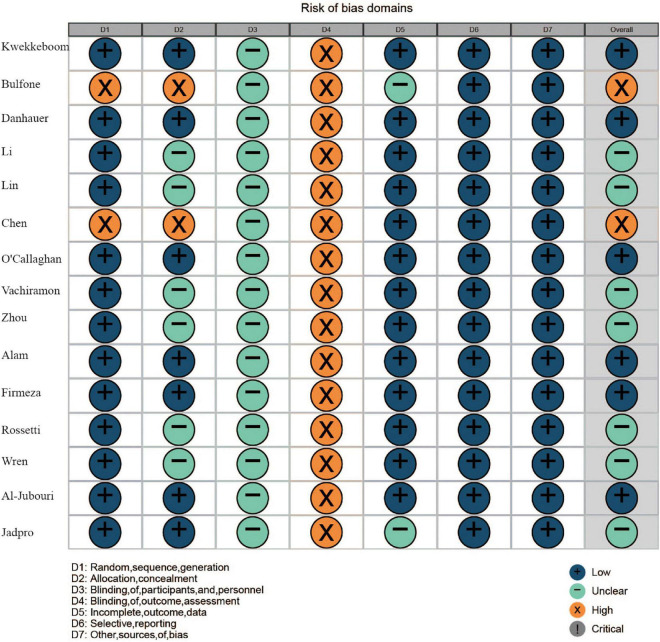
Risk of bias assessment for 15 included studies.

The risk of bias assessment result is shown in Figure 2. Two trials did not report the randomized allocation in the procedure, thus allocation concealment was also considered as “high risk,” as they did not display the randomization procession method. As the performance bias was not avoided for music therapy, all 15 trials were deemed as “unclear risk” in the blinding of participants and personnel. The detection bias was unclear for all trials. Thirteen trials reported attrition (dropout of participants) in the experiment, and they were evaluated as “low risk” in the attrition bias aspect. No reporting and other biases were observed in all trials.

### 3.3. Performance of music therapy

**FIGURE 3:**
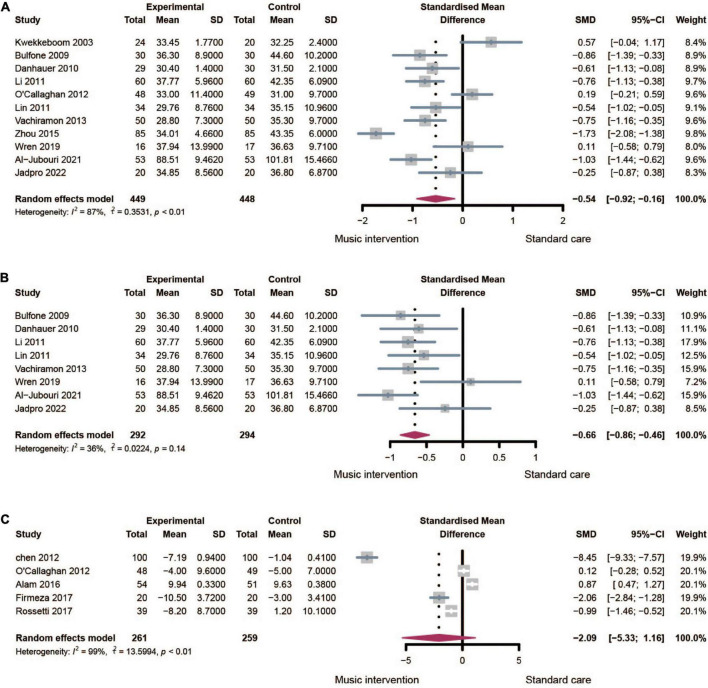
Forest plots for music therapy on anxiety. **(A)** Effect of music therapy measured by post-treatment anxiety with high heterogeneity. **(B)** Effect of music on therapy measured by post-treatment anxiety after sensitivity analysis. **(C)** Effect of music on therapy measured by anxiety changes.

Eight trials reported the anxiety score in post-treatment measured by STAI. No significant difference in anxiety baseline was observed in these trials before the music therapy was performed. As high heterogeneity was observed (*I*^2^ = 68%), the random effects model was applied to the analysis. The meta-analysis (Figure 3A) showed a positive combined effect in favor of music therapy (SMD: −0.54, 95% CI: [−0.92, −0.16], *I*^2^ = 87%). Sensitivity analysis (Figure 3B) indicated that the three studies extremely affected the overall heterogeneity. By omitting these studies, low heterogeneity was observed. In addition, the pooled effect of music invention was moderate (SMD: −0.66, 95% CI: [−0.86, −0.46], *I*^2^ = 36%).

Five trials displayed anxiety score changes measured by STAI. A random-effects model was performed as the high heterogeneity (*I*^2^ = 99%) in the analysis. The meta-analysis (Figure 3C) demonstrated that music therapy could alleviate anxiety in patients with cancer (SMD: −2.09, 95% CI: [−5.33, −1.16]). Sensitivity analysis indicated that high heterogeneity was unavoidable by omitting any one trial. Moreover, given the limited trial number, subgroup analysis was not suitable.

### 3.4. Subgroup analysis

#### 3.4.1. Gender distribution

**FIGURE 4:**
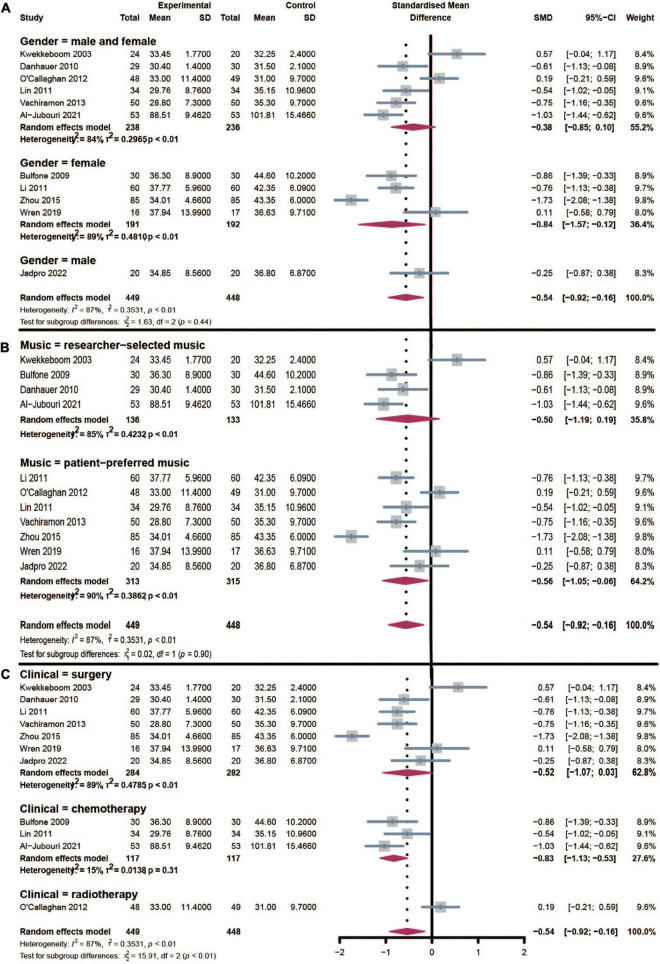
Subgroup analysis of the effect of music therapy on gender distribution **(A)**, music type **(B)**, and clinical procedure **(C)**.

The subgroup analysis of gender distribution was displayed in Figure 4. Studies could be divided into the female, male, and mixed groups in association with gender. Considering there was only one study in the male group, we compared the female and mixed groups in the below analysis. The effect of music therapy in the mixed group was calculated with an SMD of −0.38 (95% CI: [−0.85, 0.10], *I*^2^ = 84%). Whereas, a greater effect was calculated in the female group (SMD: −0.84, 95% CI: [−1.57, −0.12], *I*^2^ = 89%). Since a decrease in inter-study heterogeneity was not observed, gender distribution may not be the source of heterogeneity.

#### 3.4.2. Music type

According to the music type provided to patients with cancer, studies could also be divided into researcher-selected music, and patient-preferred music groups (Figure 4B). Music therapy could significantly reduce treatment-related anxiety in the researcher-selected music group (SMD: −0.50, 95% CI: [−1.19, 0.19], *I*^2^ = 85%). And patient-preferred music offered a similar positive effect in anxiety reduction (SMD: −0.56, 95% CI: [−1.05, −0.06], *I*^2^ = 90%). Similarly, the inter-study heterogeneity was still high in these groups, and music type was not a potential moderator variable that caused heterogeneity.

#### 3.4.3. Clinical procedure

All the included studies were performed to explore the effect of music therapy in reducing treatment-related anxiety. These clinical procedures included surgery, chemotherapy, and radiotherapy (Figure 4C). Music intervention could significantly alleviate surgery-related anxiety with an SMD of −0.52 (95% CI: [−1.07, 0.03], *I*^2^ = 89%). Whereas, music would have a greater effect in reducing chemotherapy-related anxiety (SMD: −0.83, 95% CI: [−1.13, −0.53], *I*^2^ = 15%) with a much lower heterogeneity. These factors indicated that clinical procedure was mainly or partly the source of heterogeneity. As only one study was in the radiotherapy group, subgroup analysis was not available for it.

#### 3.4.4. Cancer type

Four studies were performed among patients with breast cancer. Music therapy (Supplementary Figure 1) could significantly reduce treatment-related anxiety in breast patients with breast cancer (SMD: −0.84, 95% CI: [−1.57, 0.12], *I*^2^ = 89%).

### 3.5. Publication bias

The publication bias of the included studies was displayed in Supplementary Figure 2. The relatively symmetrical funnel plot indicated that there was no publication bias among all the included studies.

## 4. Discussion

The effectiveness of music intervention on cancer-related anxiety is disputable. In the present study, a comprehensive literature search was performed to synthesize novel randomized controlled trials to provide high-quality evidence in this aspect. The meta-analysis results indicated the positive effect of music intervention on anxiety management during routine clinical treatment among patients with cancer covering chemotherapy, radiation therapy, and surgery without general anesthesia.

A moderate superiority of anxiety alleviation in music intervention (SMD: −0.54, 95% CI: [−0.92, −0.16]) was observed compared with standard care. Inconsistent with the initial meta-analysis published in 2013 (Nightingale et al., 2013), the result supported the positive impact of music therapy on treatment-related anxiety. The contradiction was mainly derived from the included limited studies (four RCTs). Two previous meta-analysis reviews also reported the combined effect of music on anxiety reduction. Li et al. (2020) included six RCTs with anxiety score changes as the outcome data. On the contrary, Nguyen et al. (2022) used post-test anxiety scores in another six RCTs. The drawback of these studies above was the insufficient number of included literature works. Moreover, the two previous pooled studies included diverse scales to measure the baseline and post-intervention anxiety levels, including State-Trait Anxiety Inventory State (STAI-S), the Self-Rating Anxiety Scale (SAS), and the Hospital Anxiety and Depression Scale (HADS) tests. This would lead to potential bias in the literature selection.

In the present study, we systematically searched all the relevant works of literature which reported anxiety score changes or post-test anxiety score levels. To avoid potential bias and heterogeneity, we further restricted to enroll RCTs involving adults and measured by STAI, which was commonly used for medical patients. In total, 15 randomized controlled trials were included. In our opinion, anxiety score change would be more suitable for quantifying the effect of music therapy. Although all the studies declared that the baseline anxiety level was similar (*P* < 0.05) between the intervention group and the controlled group. Considering diverse cancer types, various stages, gender, or age distribution in different studies, baseline anxiety levels and post-test scores were greatly discrepant among studies which would also increase potential heterogeneity in the meta-analysis. To inspire subsequent RCTs to improve experimental design, we did not exclude four studies that only displayed the anxiety score change, and they were analyzed separately. The SMD method was also used in this study to synthesize the data.

Yet, the present study observed high heterogeneity (*I*^2^ = 87%) in the data synthesis which should be interpreted with caution. Subgroup analysis was performed to seek the potential factors of high heterogeneity. A previous study reported that women and the elderly would be more likely to suffer from anxiety after diagnosis of cancer (Linden et al., 2012). In our subgroup of gender distribution, music therapy would be more effective in the female group compared with the mixed group in association with gender. It indicated that non-pharmacological interventions like music therapy were suitable for routine clinical practice. Subgroup analysis revealed that gender distribution would not affect heterogeneity in the analysis. In the subgroup analysis of music type, the performance of researcher-selected music and patient-preferred music seemed similar. More high-quality RCTs were still needed to support this conclusion. Similarly, heterogeneity was not caused by music type. In the subgroup of clinical procedure, we revealed that music therapy would be more effective in reducing chemotherapy related with low heterogeneity (*I*^2^ = 15%). It indicated that different clinical procedures in the included studies were the source of the heterogeneity. Meanwhile, we noticed high heterogeneity in the surgery group. The diverse surgery category in the included studies would be the reason such as mastectomy, prostatectomy, bone marrow biopsy, port placement, and Mohs surgery. Considering the limited number of RCTs, more specialized subgroups were not available.

To reduce high heterogeneity, sensitivity analysis was also performed. It indicated that there was low heterogeneity (*I*^2^ = 36%) when omitting the three studies (Kwekkeboom, 2003; Zhou et al., 2015; Wren et al., 2019). Besides, several factors would also impact the heterogeneity in this study. Due to the limited number of included studies, we could not use subgroup analysis to explore the potential impact of age, cancer type, cancer staging, duration of music, or frequency of intervention. All the abovementioned discrepancies would be unavoidable, leading to heterogeneity in this study. More pieces of evidence of high quality in future were needed.

In terms of risk of bias, the majority of included RCTs lacked adequate quality and were deemed high risk in the bias assessment. Two trials did not describe the allocation concealment procedure. As music inventions were not suitable for participant blindness, all the trials were considered as an unclear risk in the performance bias. None of the trials adopted blinding of outcome assessment in the experiment design. Future studies should optimize the experiment design to obtain more high-quality results.

This meta-analysis has some primary limitations: First, the number of included studies is limited which may unavoidably result in bias and high heterogeneity. Another limitation was that the quality of the included studies was moderate. Besides, unpublished studies, including “gray studies,” were not included in our meta-analysis. These factors may have a negative impact on the results of our study.

## 5. Conclusion

The present analysis demonstrated that music intervention could reduce cancer-related anxiety with a moderate effect. However, considering the high heterogeneity and risk of bias of the included trials, more well-performed and larger-scale RCTs are required to verify the value of music intervention in reducing anxiety among patients with cancer.

## Data availability statement

The original contributions presented in this study are included in the article/Supplementary material, further inquiries can be directed to the corresponding author.

## Author contributions

LZ: conceptualization and writing—original draft preparation. CC: methodology and software. YZ: data curation and visualization. XL: writing—review and editing and supervision. All authors contributed to the article and approved the submitted version.
