# Supplementary material for: Music therapy effect on anxiety reduction among patients with cancer: A meta-analysis
Source: Front Psychol. 2023 Jan 6;13:1028934. doi: 10.3389/fpsyg.2022.1028934 (PMC9853974; doi:10.3389/fpsyg.2022.1028934)
Supplement: Supplementary file 1 [file Presentation_1.pdf]

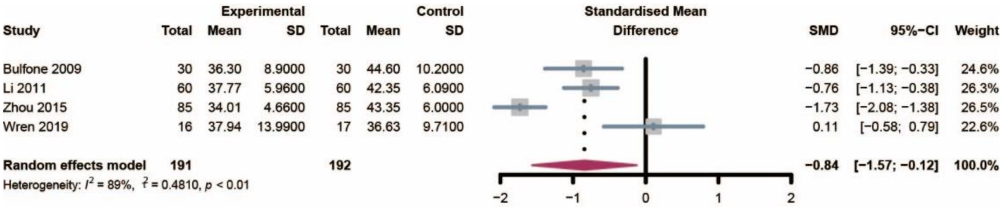

Supplementary Figure 1. Forest plots of the effect of music therapy in breast cancer patients.

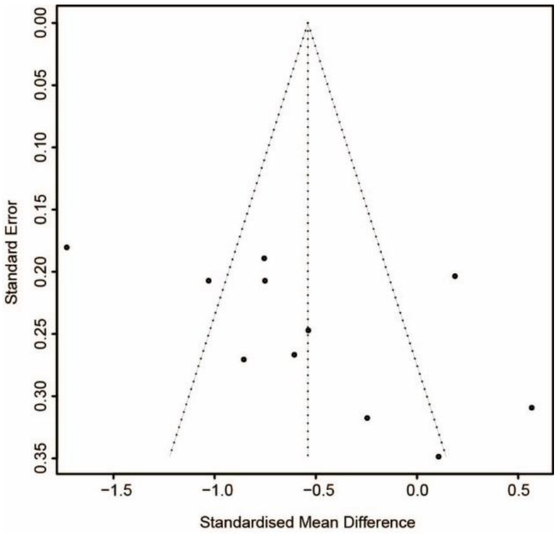

Supplementary Figure 2. Funnel plot of the included studies
